# Comprehensive Analysis of Foulants in an Ultrafiltration Membrane Used for the Treatment of Bleach Plant Effluent in a Sulfite Pulp Mill

**DOI:** 10.3390/membranes11030201

**Published:** 2021-03-12

**Authors:** Gregor Rudolph, Basel Al-Rudainy, Johan Thuvander, Ann-Sofi Jönsson

**Affiliations:** 1 Department of Chemical Engineering, Lund University, P.O. Box 124, SE-221 00 Lund, Sweden; basel.al-rudainy@chemeng.lth.se (B.A.-R.); ann-sofi.jonsson@chemeng.lth.se (A.-S.J.); 2 Department of Food Technology, Engineering and Nutrition, Lund University, P.O. Box 124, SE-221 00 Lund, Sweden; johan.thuvander@food.lth.se

**Keywords:** membrane fouling, lignin, hemicelluloses, polymeric membrane, ultrafiltration, magnesium

## Abstract

Fouling is a major obstacle in the introduction of membrane processes in new applications in the pulping industry. Due to the complex nature of the feed solutions, complementary analysis methods are usually needed to identify the substances involved. Four different methods were used for the comprehensive analysis of a membrane removed from an ultrafiltration plant treating alkaline bleach plant effluent in a sulfite pulp mill to identify the substances causing fouling. Magnesium was detected both on the membrane surface and in the nonwoven membrane backing and a small amount of polysaccharides was detected after acid hydrolysis of the fouled membrane. This study provides information on foulants, which can be used to improve processing conditions and cleaning protocols and thus the membrane performance in pulp mill separation processes. It also provides an overview of the usefulness of various analytical methods.

## 1. Introduction

Membrane filtration has already established itself as a highly selective and energy-efficient separation process in various industries [1,2], among them pulp and paper mills [3,4]. One of the main challenges in membrane processes is that the membrane capacity decreases with time due to membrane fouling. The flux can often be recovered by chemical membrane cleaning. The pure water flux after cleaning is usually used to evaluate whether cleaning has been successful [5]. Unfortunately, this is not a reliable method, as recovery of the pure water flux does not guarantee that the fouling has been completely removed [6] and the membrane capacity fully recovered. In order to demonstrate that cleaning has been successful, it is necessary to investigate the membrane ex situ to ascertain whether all the deposits on the surface and inside the membrane have been removed. In designing an efficient cleaning procedure, it is thus necessary to identify the chemical compounds deposited on and adsorbed in the membrane. What makes it especially challenging is that the deposits encountered in membrane processes in the pulping industry usually consist of a complex mixture of substances, requiring the use of several analytical methods to detect them. Moreover, the functional group characteristics of the membrane itself often interfere with those of the foulants during characterization [7].

Complementary methods of analysis must be used to characterize membrane fouling comprehensively in order to obtain information on the structure and chemical composition of the foulants. The methods typically used for ex situ analysis of membrane fouling in the pulping industry are scanning electron microscopy (SEM) [8,9,10,11,12] and attenuated total reflection-Fourier transform infrared spectroscopy (ATR-FTIR) [8,10,11,12,13]. SEM has previously been used to analyze ultrafiltration (UF) membranes fouled by thermomechanical pulp mill process water [8], and to investigate the effects of the operating conditions on fouling during nanofiltration of pulp and paper mill wastewater [9]. It has also been applied to study the efficiency of different cleaning protocols for membranes fouled by thermomechanical pulp mill process water [10] and fouling by alkaline peroxide mechanical pulping effluent [11]. SEM in combination with energy dispersive X-ray spectrometry (SEM-EDS) provides information not only on the structure, but also on the elemental composition of the fouling layer and is already commonly used for the characterization of fouling in desalination processes [12,14,15]. Nuclear magnetic resonance spectroscopy (NMR) has also recently been used to analyze chemical compounds in lignocellulosic biorefining, and has been used, for example, to obtain structural information on the antisolvent precipitates of spent sulfite liquor [16] and to characterize the low molecular weight fraction after membrane filtration of Kraft lignin [17]. Foulant extraction from the membrane, or hydrolysis of the fouling layer and subsequent analysis of the liquid fraction by liquid or gas chromatography is another approach used to obtain information on the composition of foulants. Gas chromatography has been used, for example, to identify the extractives fouling a membrane used for UF of pulp and paper process water [12,13] and liquid chromatography to determine the polysaccharide content of UF membranes fouled by thermomechanical pulp mill process water [8].

The aim of this study was to identify the foulants causing severe flux decline during UF of alkaline bleach plant effluent from sulfite pulping and to provide an overview of the usefulness of various analytical methods. A number of analytical methods were used to obtain broad information about the foulants in and on the membrane. Alkaline membrane cleaning was applied to gain additional insight. The structure of the membrane surface and the content of different elements in the fouling layer and the membrane were studied using SEM-EDS. The functional group characteristics of the membrane and the foulants were investigated with ATR-FTIR. Heteronuclear single quantum coherence 2D NMR (HSQC-2D-NMR) was used to identify functional group characteristics after the extraction of the foulants from the membrane samples with an acetone-water solution. High-performance anion-exchange chromatography coupled with pulsed amperometric detection (HPAEC-PAD) was used to determine the content of polysaccharides in the hydrolysate after hydrolyzing the fouling layer with sulfuric acid. The membrane studied in this work was used at the UF plant at the Stora Enso Nymölla sulfite pulp and paper mill in Nymölla, Sweden treating alkaline bleach plant effluent. The plant has been in operation since 1995, and is the largest of its kind in the world [18,19]. The UF plant is divided into two separate lines, a softwood line and a hardwood line. The membranes studied in this work were from the softwood line. Our findings can be used to improve the filtration process and to optimize membrane cleaning in this UF plant.

## 2. Materials and Methods

The total dry solids content of the alkaline bleach plant effluent was 16.9 mg/g, of which inorganic substances make up the largest part, 11.2 mg/g. The total lignin content of the effluent, measured with UV/vis spectroscopy at 280 nm, was 4.2 mg/g. The UF plant on the softwood line at the Stora Enso Nymölla pulp and paper mill contains seven stages equipped with 3.6 m long tubular PCI ES404 membranes (PCI Membranes, now part of Filtration Group, Hampshire, UK) made of polyether sulfone, with a cut-off of 4 kDa. The softwood UF plant consists of seven stages with a total membrane area of 2900 m^2^. The module inlet pressure is 7 bar and the cross-flow velocity is 1.8 m/s. A brief description of the plant can be found in [19]. Fouled membranes were collected when the membranes in the first stage of the softwood line were replaced. One membrane tube was rinsed with deionized water, and samples of the rinsed membrane were subsequently cleaned with an alkaline cleaning agent for 1 h or for 20 h, before being analyzed. The results obtained from the rinsed and cleaned membrane samples were compared with those from a pristine membrane used as reference.

### 2.1. Experimental Procedures

Preservatives in the pristine membrane were removed by cleaning with deionized water in an ultrasonic bath. The membrane was cut into 10 cm long tubes and sonicated 3 times for 10 min, with intermediate water changes. The fouled membrane was also cut into 10 cm long tubes and rinsed with deionized water. Six of the tubes were divided in half lengthwise and mounted in a holder by inserting one end of each membrane half-tube between two pieces of plastic held together by metal screws. The membranes were then suspended in a beaker containing 2500 mL of a 2% alkaline solution of BanUltra 17 (Banmark, Vantaa, Finland). The cleaning solution was agitated using an electric stirrer, and the temperature was maintained at 50 °C using a temperature-controlled heating plate. After 1 h, half of the membrane half-tubes were removed, rinsed in deionized water and stored in a beaker containing deionized water before analysis. Cleaning of the remaining six membrane half-tubes continued for a total of 20 h, after which the membrane half-tubes were rinsed with deionized water and stored in a beaker with deionized water before analysis. When preparing the samples for SEM-EDS analysis, plastic zip-ties were used to hold the membrane samples instead of metal screws, in order to reduce the exposure of the membrane samples to metal during cleaning. Cleaning was otherwise performed as described above.

### 2.2. Analysis

Membrane samples were analyzed using SEM-EDS and ATR-FTIR. The content of hemicelluloses was determined by acid hydrolysis of the membrane samples and analysis of the hydrolysate with HPAEC-PAD. The functional group characteristics of the bleach plant effluent and substances extracted from pristine, fouled, and cleaned membrane samples were determined using HSQC-2D-NMR.

#### 2.2.1. SEM-EDS Measurements

The structure of the membrane surface and the content of inorganic material were investigated using SEM in combination with EDS. Membrane half-tubes were cut into 5 × 5 mm^2^ large coupons. For cross-sectional analysis, the membranes were dipped in liquid nitrogen and then broken to create a straight cut-line. Air-dried membrane coupons were coated with a gold–palladium mixture at a current of 150 mA and a pressure of 7 × 10^−2^ mbar under an argon atmosphere for 3 min. Three positions on each coupon were examined in a JSM 6700F NT microscope (JEOL, Tokio, Japan) at an acceleration voltage of 10 kV. At least, one position on each coupon was analyzed with an EDS system (Oxford Instruments, Abingdon, UK). The spectra obtained were quantified using the AZtec v2.3 software supplied by the manufacturer. 

#### 2.2.2. ATR-FTIR Measurements

ATR-FTIR was used to determine the functional group characteristics of the membrane and deposited foulants. Membrane coupons measuring 5 × 5 mm^2^ were dried in a desiccator before analysis. The transmittance of each coupon was measured at three positions. Spectra were obtained with an ALPHA-P FTIR spectrometer (Bruker, Billerica, MA, USA), equipped with a platinum ATR sampling module with a single bounce diamond crystal. The absorbance was recorded in the wavenumber interval 400 to 4000 cm^−1^. During each measurement, 72 scans were recorded with a resolution of 4 cm^−1^. The spectra were baseline corrected and the results presented as the transmittance. To increase the contact between the membrane coupon and the crystal, measurements were also conducted with a drop of deuterium oxide (Sigma-Aldrich, Darmstadt, Germany) between the ATR-FTIR crystal and the coupon. The bleach plant effluent was also analyzed with ATR-FTIR. The effluent was lyophilized and analyzed in triplicate by ATR-FTIR with the same parameters (wavenumber interval, scans, resolution) as described above. No measurements were carried out with deuterium oxide in the analysis of the effluent.

#### 2.2.3. HPAEC-PAD Measurements

The content of polysaccharides in the membrane samples was analyzed with HPAEC-PAD. A 10 cm long membrane half-tube, corresponding to a 5 cm long tubular membrane, from a pristine membrane, a fouled membrane, and membranes cleaned for 1 h and 20 h, was analyzed. The surface area of each membrane sample was 19.9 cm^2^. The membrane half-tubes were cut into coupons and suspended in 5 mL deionized water. A volume of 188 µL of 72% sulfuric acid was added, and the membrane coupons were incubated at 120 °C for 1 h in order to hydrolyze polymeric sugars to their respective monomers. The membrane coupons were removed and the sugar monomers in the remaining solution were analyzed using HPAEC-PAD in an ICS-3000 chromatography system (Thermo Fisher Scientific, Waltham, MA, USA). Deionized water was used as the eluent at a flow rate of 1 mL/min, with a post-column addition of 0.5 mL/min of 200 mM NaOH. d-Galactose, d-glucose, d-mannose, d-xylose, and l-arabinose (Fluka Chemie, Buchs, Switzerland) were used as standards.

#### 2.2.4. HSQC-2D-NMR Measurements

HSQC-2D-NMR was used to determine the functional group characteristics of substances dissolved during the extraction of membrane samples. For each sample, membrane half-tubes corresponding to a total of 18 cm of a tubular membrane (four pieces of 9 cm long halved tubes, 72 cm^2^ membrane area) were cut into coupons and extracted using a Soxhlet extractor. An 1:9 acetone-deionized water mixture, by volume, was used for the Soxhlet extraction as this mixture has been demonstrated to be efficient to recover organic foulants on membranes treating pulp mill effluent [20]. A total volume of 190 mL was used for each coupon. The membranes were extracted for 20 h. After extraction, the membrane coupons were discarded, and the solution containing the extracted foulants was collected. The solvent was then evaporated and the remaining solids were dried in a desiccator. These solids were subsequently re-dissolved in 1 mL of deuterium oxide, where the solvent served as the internal reference with the chemical shifts of δ_C_/δ_H_ = −4.81 ppm. The instrument used was a Bruker Avance III HD 500 MHz spectrometer (Bruker, Billerica, MA, USA), which was equipped with a 5 mm Broadband Observe Probe (PABBO BB/19F-1H/D) and a Z-gradient coil (Z-GRD Z119470/0142). Data were acquired with the pulse program “hsqcetgpsisp.2” using the following settings: 1.0 s relaxation delay, 9.5 µs pulse length, spectral width of 11 ppm, FID size 1538 and a total of 136 scans. The data were baseline- and phase-corrected with MestReNova 12 (Mestrelab Research, Santiago de Compostela, Spain).

## 3. Results and Discussion

### 3.1. Visible Appearance

**Figure 1:**
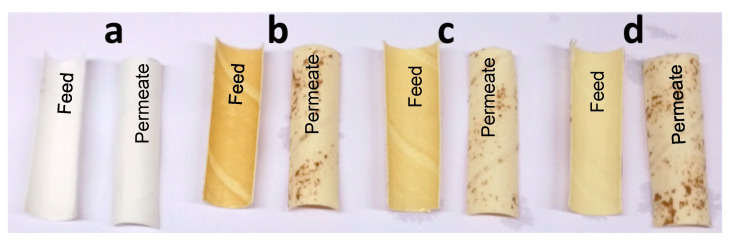
Visual appearance of the feed side and permeate side of: (**a**) a pristine membrane, (**b**) a fouled membrane, (**c**) a membrane cleaned for 1 h with the alkaline cleaning agent, and (**d**) a membrane cleaned for 20 h with the alkaline cleaning agent.

Before analysis of the membrane samples, their visible appearance was documented. The fouled membrane had a noticeably different appearance from the pristine membrane, in that it had an even, brownish color on the feed side of the membrane, as can be seen in Figure 1. The discoloration of the membrane surface on the feed side of the membrane was gradually removed during cleaning. Distinct brown spots were observed on the surface of the nonwoven backing on the permeate side. The brown spots appeared to be on the outer surface of the backing material on the permeate side. No visible change was observed in the brown spots after cleaning. 

### 3.2. Surface Structure and Content of Elements Determined with SEM-EDS

**Figure 2:**
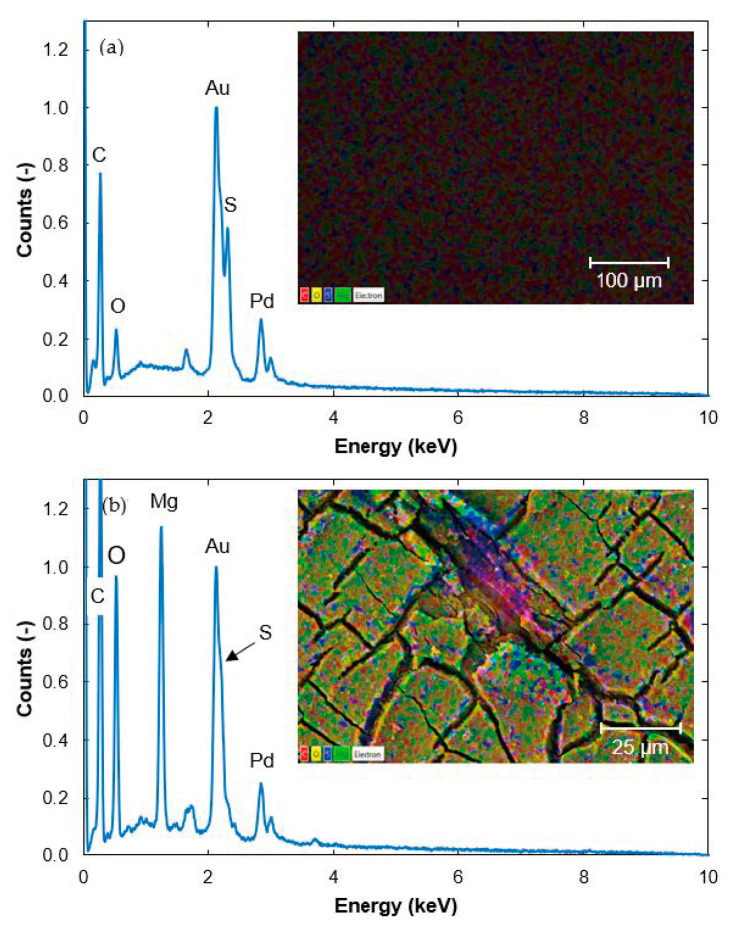
Energy dispersive X-ray spectrometry (EDS) spectra from: (**a**) a pristine membrane and (**b**) a fouled membrane. Scanning electron microscopy (SEM) images of the membrane surface are shown in the inserts. Colors in the images indicate carbon (red), oxygen (yellow), sulfur (blue) and magnesium (green). The values on the *y*-axis are normalized to the value of the Au peak at 2.13 keV.

A significant difference was seen between the SEM images of the surface of a pristine membrane and a fouled membrane. The surface of the pristine membrane was smooth and homogeneous (Figure 2a), while the fouling on the surface of the fouled membrane was cracked (Figure 2b, note the different scale). The fouling layer probably cracked when the membrane was dried in the desiccator before the SEM analysis, or in the vacuum environment in the SEM instrument.

As the membrane samples were coated with a mixture of gold (Au) and palladium (Pd) before SEM imaging, these were detected in all samples during EDS. Other peaks seen in the EDS spectrum of the pristine membrane were from sulfur (S), carbon (C), and oxygen (O) (Figure 2a), which are expected to be found in polyethersulfone membranes. The dominant color of the fouled membrane (Figure 2b) was green, indicating that the fouling layer consisted mainly of magnesium (Mg). Mg originates from the cooking chemical base, which is Mg at the pulp and paper mill. Silicon (Si) and calcium (Ca) were also detected on the fouled membrane but at counts below the quantification limits (Figure 2b). These elements originate from the wood. Mg, Si, and Ca have also been detected previously on fouled membranes from the pulp and paper mill [21].

#### 3.2.1. Contents of Elements in a Cross-Section of the Fouled Membrane

**Figure 3:**
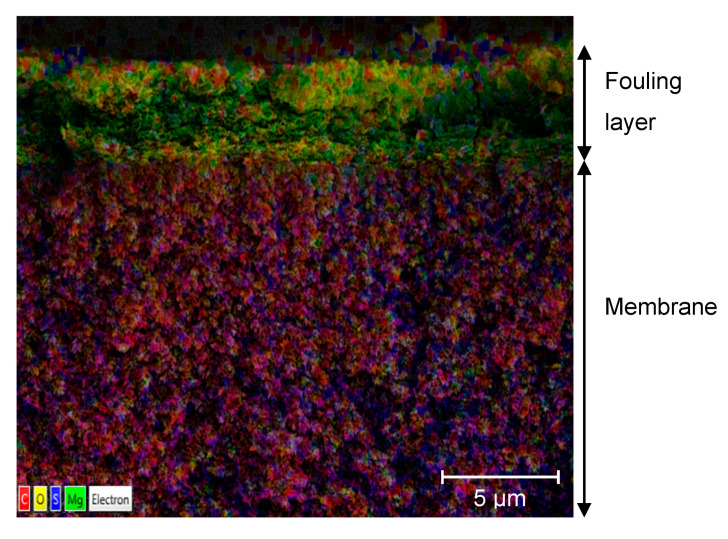
SEM-EDS image of the cross-section of the fouling layer and the active membrane layer of the fouled membrane.

Figure 3 shows a cross-section of a fouled membrane sample. The fouling layer on the membrane surface is 4–5 μm thick and contains mainly Mg (green spots) but also C (red), and O (yellow). The membrane active layer contains mainly C (red) and S (blue), and some O (yellow).

**Figure 4:**
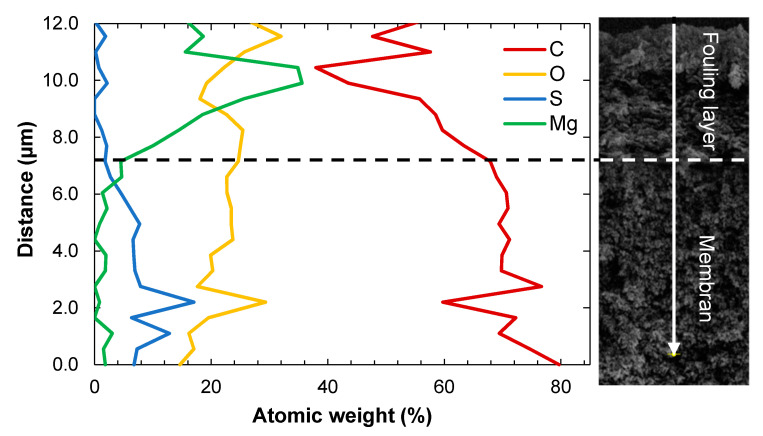
The atomic weight % of different elements in the cross-section along the fouling layer and active layer of the membrane (**left**) and SEM image of the cross-section of the fouling layer and the active layer of the membrane (**right**).

Figure 4 shows the contents of different elements in active layer of the membrane and the fouling layer. Small amounts of S were detected in the fouling layer. These could be related to lignosulfonates or the remains of cooking chemicals such as SO_2_. The fouling layer seems to consist of two parts: the part closest to the membrane surface containing mostly Mg and Si, and the upper part where C and O dominate, indicating the presence of organic substances. The amount of C is reduced in towards the end of the fouling layer and the beginning of the membrane active layer. It appears that the amount of Mg is highest on top of the membrane and that a notable amount of Mg penetrates the first 1–2 µm of the membrane.

#### 3.2.2. Contents of Elements in the Non-Woven Backing

**Figure 5:**
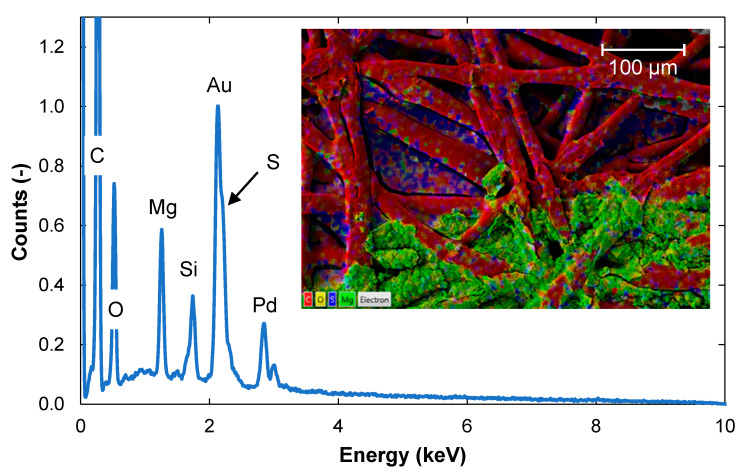
EDS spectrum obtained from the backing of the fouled membrane. A SEM image of the membrane surface is shown in the insert. The elements present and normalization are as described in Figure 2.

Foulants were also found in the non-woven backing of the membrane (seen as brown spots in Figure 1). These precipitates were only found in certain areas, while other areas were completely clean. An example of such a fouled area is shown in Figure 5, where it can be seen that the spots contained Mg, Si, C, and O. 

#### 3.2.3. Removal of Foulants by Cleaning

**Figure 6:**
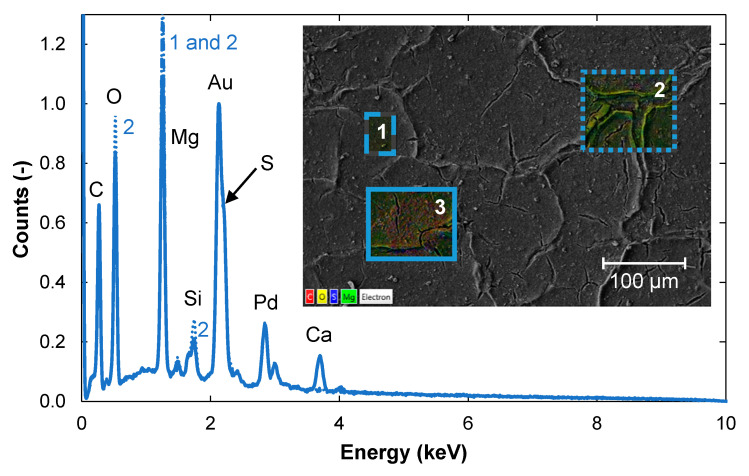
EDS spectrum from three areas (1 = solid line, 2 = dotted line, and 3 = dashed line) on a membrane sample that had been cleaned for 1 h. A SEM image of the membrane surface is shown in the insert. EDS analysis was carried out on the three areas indicated in the SEM image. The elements present and normalization are as described in Figure 2.

SEM analysis showed that the fouling layer was not completely removed after 1 h of cleaning (Figure 6). EDS analysis at three positions (1, 2 and 3) on the membrane sample showed that the quantities of the various elements varied at different locations. The dominant elements of the fouling layer were Mg, O, and C. Small amounts of Si were detected in all three areas, while Ca was only detected in area 3. In the pulp mill, calcium carbonate is used for the fine-tuning the paper with regard to, among other things, opacity, whiteness and bulk properties [22]. It might have that a solution with calcium carbonate dropped on the sample when the membrane was taken from the plant.

**Figure 7:**
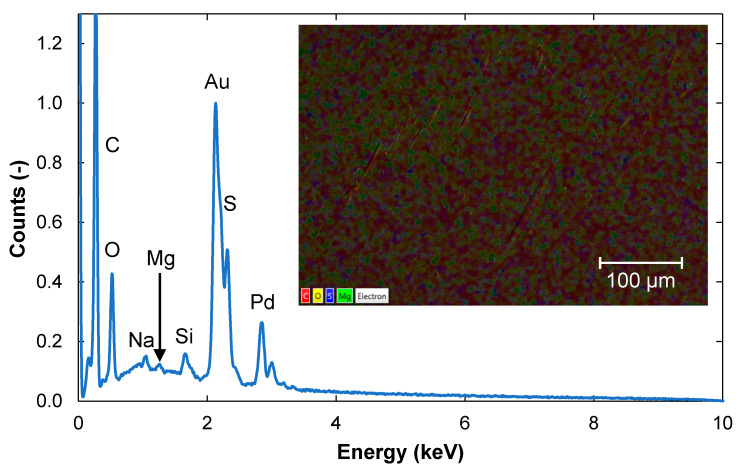
EDS spectrum of a membrane sample that had been cleaned for 20 h. A SEM image of the membrane surface is shown in the insert. The elements present and normalization are as described in Figure 2.

After 20 h of cleaning, the fouling layer seemed to have disappeared (Figure 7), and the appearance of the surface was very similar to that of the pristine membrane (Figure 2). However, remains of Si and Mg were still detectable on the membrane surface. It is likely that acidic cleaning would have removed these remaining foulants, but this was out of the scope of this work.

**Figure 8:**
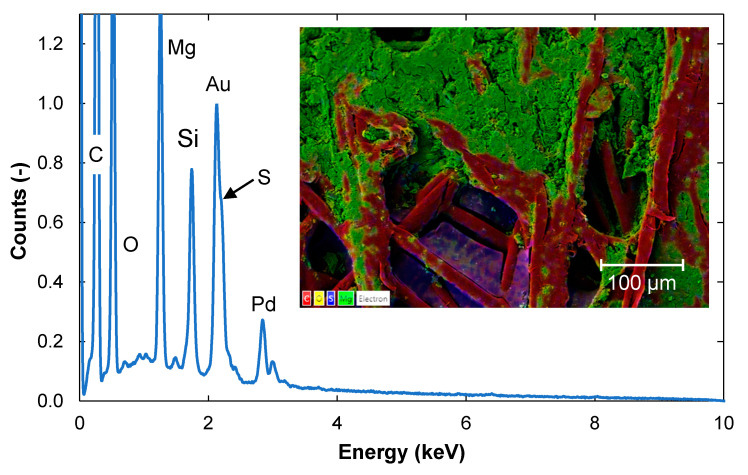
EDS spectrum of the backing of a membrane sample that had been cleaned for 20 h. A SEM image of the membrane surface is shown in the insert. The elements present and normalization are as described in Figure 2.

In contrast to the feed side of the membrane, where most Mg was removed from the surface after 20 h cleaning (Figure 7), a significant amount of Mg and Si remained in the backing of the membrane (Figure 8). 

### 3.3. Identification of Foulants by ATR-FTIR

**Figure 9:**
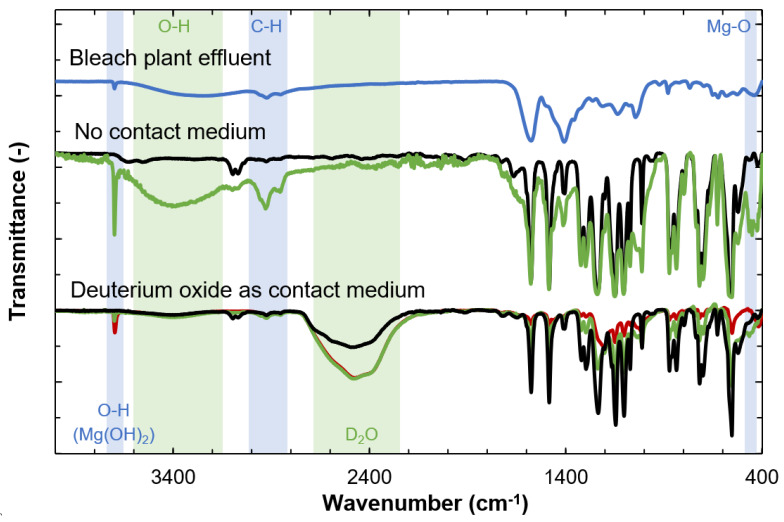
Attenuated total reflection-Fourier transform infrared spectroscopy (ATR-FTIR) spectra obtained from: lyophilized bleach plant effluent (**blue line**), a pristine membrane (**black line**), a membrane cleaned for 1 h (**red line**) and a membrane cleaned for 20 h (**green line**). The contact between the ATR crystal and the sample was increased by using deuterium oxide as contact medium. No spectra were obtained for the membrane cleaned for 1 h when not using any contact medium but was successful when using deuterium oxide as contact medium. The spectra are averages of three membrane coupons.

ATR-FTIR was used to identify organic substances by providing information on the chemical bonds detected. Despite repeated analysis, no peaks were observed in the spectra from the fouled membrane or that cleaned for 1 h. This was probably due to insufficient contact between the membrane coupons and the ATR crystal. A drop of deuterium oxide was therefore applied to the crystal to improve the contact between the membrane coupon and the crystal. It was then possible to obtain a spectrum from the membrane cleaned for 1 h, but it was still not possible to obtain an adequate signal from the fouled membrane. Spectra could be obtained from the pristine membrane and the membrane cleaned for 20 h. The spectra obtained are shown in Figure 9. The only visible difference between the membranes cleaned for 1 h and 20 h was a slightly more pronounced peak at 3668 cm^−1^.

The broad signal in the measurements made without the contact medium in the interval 3200 to 3600 cm^−1^ in Figure 9 could indicate the presence of O–H groups, which are present in both lignin and polysaccharides [23]. However, since this peak is absent in the spectra when deuterium oxide was used as contact medium, the broad signal probably originates from the presence of water in the sample. In the spectra obtained from the samples where deuterium oxide was used as a contact medium, a prominent peak was observed at 2461 cm^−1^, which is ascribed to the deuterium oxide [24].

The presence of Mg was confirmed by the peaks at 3668 cm^−1^ and 423 cm^−1^, both with and without deuterium oxide. The sharp peak at 3668 cm^−1^ is attributed to the O–H bond stretching vibration in the crystal structure of Mg(OH)_2_, and the peak at around 423 cm^−1^ is assigned to the Mg-O stretching vibration in Mg(OH)_2_, according to [25] who analyzed Mg(OH)_2_ with FTIR. The peak at 2930 cm^−1^ is prominent in the spectra obtained without contact medium, but insignificant in those where deuterium oxide was used as contact medium. This could be caused by the stretching of C–H bonds in the methyl and methylene groups [23,26,27,28], and can be associated with lignin, phenolic compounds such as wood extractives, or other organic compounds.

Most peaks at wavenumber below 1800 cm^−1^ can be assigned to chemical bonds in the membrane material. The peaks of the cleaned membranes in the fingerprint region were smaller than those in the pristine membrane, confirming that the membrane is still shielded by a fouling layer even after cleaning. It was not possible to identify any specific functional group in the fingerprint region in the spectra from the cleaned membranes that differed from peaks found in the pristine membrane. Therefore, the bleach plant effluent was also analyzed with ATR-FTIR in order to identify any similarities between the bleach plant effluent and the membrane samples. However, the spectra from the membrane samples (Figure 9) and the bleach plant effluent (Figure 9) both lacked the bands characteristic of aromatic ring vibrations centered around 1600 cm^−1^ and 1500 cm^−1^. These usually appear as a pair of band structures often with some splitting [23,29]. The peak at 1500 cm^−1^ seen in the spectra from both the feed and the membrane samples could be due to C=C bonds in other substances in the bleach plant effluent, such as extractives [30].

### 3.4. Polysaccharides Detected by HPAEC-PAD

Small amounts of polysaccharides, primarily glucans, were found on the membranes, as can be seen from Table 1. This was also indicated from the ATR-FTIR analysis. Only glucans were present at quantifiable amounts. The amount of poly–saccharides was reduced by 30% and 40% after 1 h and 20 h of cleaning, respectively.

### 3.5. Identification of Foulants with HSQC-2D-NMR 

**Figure 10:**
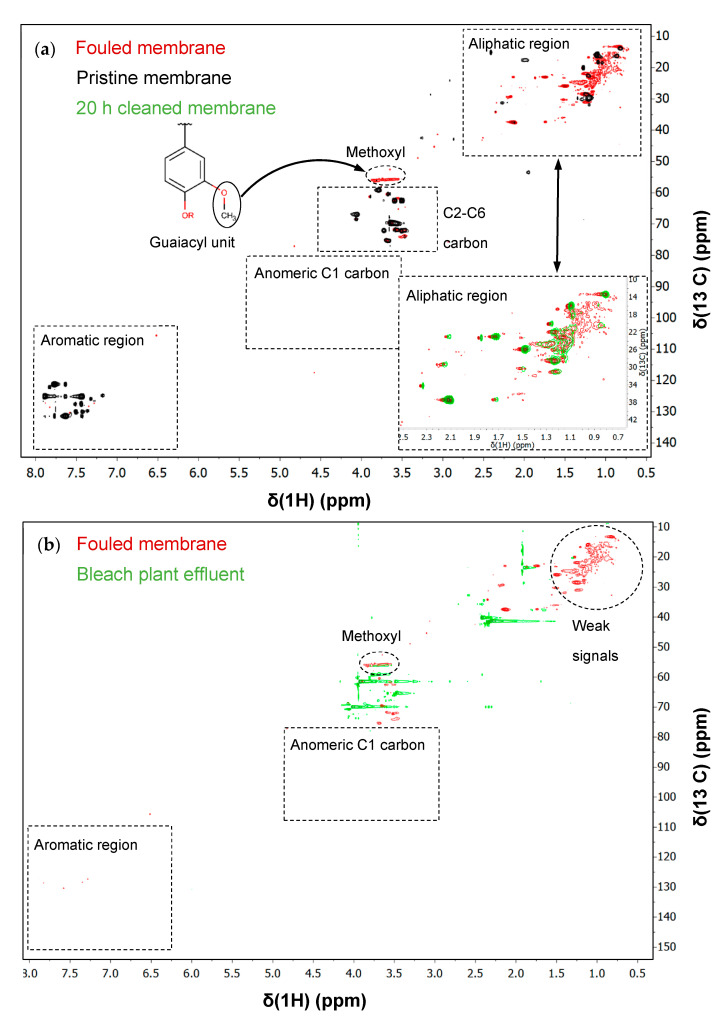
Results of heteronuclear single quantum coherence 2D nuclear magnetic resonance (HSQC-2D-NMR) measurements on: (**a**) the fouled membrane (**red**), the pristine membrane (**black**), and the membrane cleaned for 20 h (**green**), and on (**b**) the fouled membrane (**red**) and the bleach plant effluent (**green**).

Signals from the benzene ring of lignin were expected in the aromatic region (δ_C_/δ_H_: 100–140/6–8) (Figure 10). Only a few signals were detected in the sample from the fouled membrane, and no signals at all in the spectrum from the bleach plant effluent (Figure 10). Difficulties in the detection of signals in this region can result from the high molecular weight of lignin and lignin-carbohydrate complexes in the feed and on the fouled membrane that affect the spin-spin relaxation time [11,16]. In contrast, numerous signals were obtained from the pristine membrane in the aromatic region, however, these were outside the range in which aromatic signals have been found for lignin in other pulping solutions (guaiacyl units, δ_C_/δ_H_: 105–125/6–8) [16]. 2D-NMR simulations with polysulfone as the molecular structure indicated that these signals were probably from the aromatic units in the membrane material.

The only signal that could be related to lignin, and which usually has a high intensity, was the signal from the methoxyl group (δ_C_/δ_H_: 55.96/3.66). This signal was only present in the spectrum from the fouled membrane and the bleach plant effluent. This is hardly surprising as the membrane material contains no methoxyl groups [31].

Since traces of glucans were detected in the fouled membrane using HPAEC-PAD, signals from the anomeric C1 carbon were expected in the region δ_C_/δ_H_: 80–110/3.0–5.0. However, no visible signal was observed in this region in any of the spectra. The reason for this could be that the extracted polysaccharides had a high molecular weight, or the concentration was too low in the acetone-water solution, making detection difficult.

Signals for the C2 to C6 carbons in polysaccharides were expected in the region with chemical shifts δ_C_/δ_H_: 60–80/3–4.5. Signals from the pristine membrane, the bleach plant effluent and the fouled membrane were seen in this region. The signals from the fouled membrane were concentrated around the methoxyl region. Signals were probably detected from the pristine membrane because fragments of the membrane material were dissolved during the extraction process with acetone and water. This means that it is not possible to determine whether there are signals from polysaccharides in the fouled membrane sample as they could be concealed/affected by signals from dissolved membrane fragments.

No signals were detected in the aliphatic region (δ_C_/δ_H_: 5.0 –50/0.0–3.0) for the pristine membrane sample. In contrast, a high number of signals were observed in this region for the feed and fouled membrane samples. Most of the signals were seen in the extract from the fouled membrane, while the signals from the bleach plant effluent were weak. These observations suggest that the fouling compounds detected in this region are deposited and concentrated on the membrane during ultrafiltration of the bleach plant effluent. Functional groups with signals in this region are quite complex, and it is therefore difficult to interpret the results. Conclusions can only be drawn regarding the compounds causing these signals. According to 2D-NMR simulations and literature data [32], a common extractive in spruce (abietic acid) has chemical shifts in the same region. It is therefore possible that these signals originate from extractives such as fatty acids and resin acids. [33] have shown that extractives give signals in the same region of the spectrum. However, degradation products from lignin were also shown to yield signals in the same region. 

#### Analysis of a Cleaned Membrane with HSQC-2D-NMR

The signal from the methoxyl group was shown to be associated with the foulants in the bleach plant effluent and is independent of the dissolution of the membrane material. This signal was therefore chosen for the quantification of foulant removal by comparing the peak volume integral of the signals for the fouled and cleaned membranes. After cleaning for 1 h, the proportion of methoxyl groups had decreased by 18%, and after 20 h the proportion had decreased by 36%.

These values indicate that cleaning removed some of the foulants. However, the intensity of the signals in the aliphatic region (δ_C_/δ_H_: 5.0–50/0.0–3.0) did not change after cleaning, as can be seen in Figure 10. The low intensity of these compounds in the bleach plant effluent (see Figure 10) suggests that these fouling compounds have a high affinity for the membrane and are not desorbed by alkaline cleaning. 

### 3.6. Remarks on the Analytical Methods Employed

This study illustrates the opportunities and limitations of different analysis methods when studying a membrane fouled by a bleach plant effluent at a sulfite pulp mill. SEM-EDS provides images of the membrane and fouling layer, as well as information on which elements are found in the membrane, and where in the membrane they are located. Magnesium, for example, was detected in the fouling layer on the feed side of the membrane and in the nonwoven backing on the permeate side. Using SEM-EDS it is also possible to visualize the variation in the number of elements in cross-sections of the fouling layer and the active membrane layer. The drawback of SEM-EDS is that only inorganic elements can be identified, although the presence of carbon and oxygen indicates that organic substances are also present.

Bands in ATR-FTIR spectra can be assigned to certain chemical bonds making it possible to identify individual compounds. However, if the solution is a complex mixture of substances, as in the case where a number of wood components are dissolved during the bleaching process, it is difficult to differentiate between the compounds. Furthermore, when studying foulants on polymeric membranes, signals from substances in the membrane interfere, making it difficult to distinguish between signals from the membrane and foulants [7], and complicating the interpretation of the spectra.

High-performance liquid chromatography is used to separate, identify, and quantify each component in a mixture. In this study, HPAEC-PAD was used to identify sugars after hydrolyzing fouled and cleaned membranes with water and sulfuric acid. Challenges with this approach are that the compounds hydrolyzed highly depends on hydrolysis. With a different procedure, other substances would probably have been detected during the HPAEC-PAD analysis. The interpretation of the results was difficult as concentrations in the hydrolysate were low (especially for the cleaned membranes). 

HSQC-2D-NMR is a highly sensitive method of determining chemical shifts that enables the determination of the structure of organic molecules in solution. Although this is a very sophisticated analytical method, problems are encountered when it is used to determine the composition of a fouling layer on a membrane. Similar to HPAEC-PAD, the analysis is highly dependent on the solvent used to extract the fouling substances. In this study, an acetone-water solution was used. If a different solvent had been used, other substances would probably have been extracted and detected in the NMR analysis. Furthermore, the extraction of substances in the membrane can interfere with the analysis and could result in overlapping signals. 

## 4. Conclusions

Using a combination of complementary analysis methods, it was possible to identify the main foulants leading to reduced performance during UF of alkaline bleach plant effluent. Valuable structural and chemical information of the membrane surface was provided with SEM-EDS and ATR-FTIR. Further chemical insights were gained with the hydrolysis of the membrane and subsequent analysis of the hydrolysate with HPAEC-PAD and the analysis of the extract from the membrane with HSQC-2D-NMR.

The investigations revealed the location of different foulants on and in the membrane structure. The results showed that a roughly 5 µm thick fouling layer existed on top of the membrane. The layer consisted mainly of magnesium and small amounts of polysaccharides. Moreover, magnesium penetrated around 1 µm into the membrane structure. Precipitated magnesium was also found in the nonwoven backing.

The thick inorganic fouling layer impeded the analysis with ATR-FTIR. Thus, it was not possible to distinguish the functional groups of the foulants from those of the membrane. ATR-FTIR was therefore only applied after alkaline cleaning of the membrane. This provided further insight and indicated that also wood extractives could be part of the fouling layer. Extraction with acetone and water followed by HSQC-2D-NMR analysis revealed the functional group characteristics in the solution, especially of aromatic compounds. The results indicated that the common foulant in pulping, i.e., lignin, was not the main source of fouling in this study, but support the theory that wood extractives, mainly fatty acids and resin acids, are involved in the formation of fouling. 

This study has provided useful information on foulants, which can be used to improve processing conditions and cleaning protocols and thus the membrane performance in pulp and paper mill separation processes, while also providing an overview of the scope of various analytical methods. In the future, membrane cleaning focusing on acidic cleaning, and the reduction of magnesium in the pulping process should be investigated.

## Figures and Tables

**Table 1:** Amount of polysaccharides (mg/m^2^) found on the various membrane samples using high-performance anion-exchange chromatography coupled with pulsed amperometric detection (HPAEC-PAD).

Sample	Arabinan	Galactan	Glucan	Xylan	Mannan
Pristine	-	-	*	-	-
Fouled	*	*	15	*	*
1 h cleaned	-	*	10	*	*
20 h cleaned	-	-	8	*	*

- Below the detection limit. * Polysaccharides present but at amounts lower than the lowest standard used.
